# MRI to assess chemoprevention in transgenic adenocarcinoma of mouse prostate (TRAMP)

**DOI:** 10.1186/1471-2342-11-21

**Published:** 2011-12-13

**Authors:** Ali S Arbab, Adarsh Shankar, Nadimpalli RS Varma, Dorrah Deeb, Xiaohua Gao, ASM Iskander, Branislava Janic, Meser M Ali, Subhash C Gautam

**Affiliations:** 1 Department of Radiology, Henry Ford Hospital, 1 Ford Place, 2F, Detroit, MI 48202, USA; 2 Department of Surgery, Henry Ford Hospital, 1 Ford Place, 4D, Detroit, MI 48202, USA

## Abstract

**Background:**

The current method to determine the efficacy of chemoprevention in TRAMP mouse model of carcinoma of prostate (CaP) is by extracting and weighing the prostate at different time points or by immunohistochemistry analysis. Non-invasive determination of volumes of prostate glands and seminal vesicles before, during and after treatment would be valuable in investigating the efficacy of newer chemopreventive agents in CaP. The purpose of this study was to determine whether *in vivo *magnetic resonance imaging (MRI) using a 3 tesla clinical MRI system can be used to follow the effect of chemoprevention in TRAMP model of mouse CaP.

**Methods:**

Mice were randomized into control and treated groups. The animals in treated group received 10 µmol/kg of CDDO, 5 days a week for 20 weeks. Animals underwent *in vivo *MRI of prostate gland and seminal vesicles by a clinical 3 Tesla MRI system just before (at 5 weeks), during and at the end of treatment, at 25 weeks. T1-weighted and fat saturation (FATSAT) multiecho fast spin echo T2- weighted images (T2WI) were acquired. Volume of the prostate glands and seminal vesicles was determined from MR images. T2 signal intensity changes in the seminal vesicles were determined by subtracting higher echo time (TE) from lower TE T2WI. Following treatments all animals were sacrificed, prostate and seminal vesicles collected, and the tissues prepared for histological staining. All data were expressed as mean ± 1 standard deviation. Two-way or multivariate analysis of variance followed by post-hoc test was applied to determine the significant differences. A p-value of <0.05 was considered significant.

**Results:**

Histological analysis indicated tumor in 100% of control mice, whereas 10% of the treated mice showed tumor in prostate gland. Both MRI and measured prostate weights showed higher volume/weight in control mouse group. MRI showed significantly higher volume of seminal vesicles in control animals and T2 signal intensity changes in seminal vesicles of control mice indicating higher number of tumor foci, which was also proven by histology.

**Conclusions:**

*In vivo *MRI is helpful in determining the efficacy of chemoprevention of prostate cancer in TRAMP mice.

## Background

According to American Cancer Society, prostate cancer is the most frequently diagnosed malignant neoplasm among men in America and is the second leading cancer in deaths. The American Cancer Society estimates that there will be 240,890 new cases of prostate cancer diagnosed and 33,720 prostate cancer specific deaths in 2011. To help solve and find a remedy for this illness, investigators in the field of prostate cancer therapy and research are using a mouse model called TRAMP (Transgenic Adenocarcinoma of Mouse Prostate). TRAMP is a transgenic mouse model of carcinoma of prostate (CaP). The mouse model has the ability to develop spontaneous tumors, which have helped study chemopreventive treatment efficacy. This model helps to predict therapeutic responses in different chemopreventive treatment strategies.

Because of the long latency period and slow progression of the preneoplastic lesions to the malignant stage, CaP is ideally suited for developing primary, secondary and tertiary preventive strategies. Thus, intervention with plant-derived polyphenolic compounds or their synthetic analogs represents a promising approach to preventing/delaying the incidence, progression, recurrence, morbidity and mortality associated with cancer of the prostate. Triterpenes or triterpenoids are members of a larger family of structurally related compounds known as cyclosqualenoids that are widely distributed in the plant kingdom [1]. Oleanolic acid and ursolic acid are naturally occurring triterpenoids that have been used in traditional medicine as anti-cancer and anti-inflammatory agents [2-4]. 2-cyano-3,12-dioxooleana-1,9(11)-dien-28-oic acid (CDDO) has also shown chemopreventive activity in animal models of liver, breast and lung cancer [5-7], but they have not been investigated for the prevention of prostate cancer.

The current method to determine the efficacy of treatments is by extracting and weighing the prostate at different time points or by immunohistochemistry analysis. Non-invasive determination of prostate glands and seminal vesicles volumes before, during and after treatment will be valuable in investigating the efficacy of different newer chemopreventive agents in prostate cancer. Magnetic resonance imagining (MRI) is often used to analyze therapeutic efficacy in pre-clinical studies [8,9]. Only few investigators have successfully viewed the images of the mouse prostate glands and its tumor by high-resolution magnetic resonance imaging [10-12]. MRI is thought to be one of the best choices among *in vivo *imaging modalities, not only because of its high resolution but also due to its ability to separate tissues based on signal intensities. Unlike human, prostate gland in mouse has different anatomy and does not remain fixed. Mouse prostate is multi-lobed (two anterior, two dorsolateral and one ventral), which surrounds urethra and moves along with urinary bladder. Moreover, tumor in TRAMP model does not remain confined within prostate lobes and spreads in seminal vesicles [11,13,14]. Therefore, lobes of the prostate as well as seminal vesicles should be analyzed by *in vivo *imaging to determine the effects of chemoprevention. The purpose of this paper is to determine whether non-invasive *in vivo *MRI can determine the effects of treatment in TRAMP model of mouse CaP.

## Methods

### Ethical Statement

Animal experiments described in the manuscript were approved by our animal care and user committee at Henry Ford Health System (protocol number 856) according to the guideline and policies of office of laboratory animal welfare (OLAW) and public health service, National Institutes of Health. All the experiments were performed according to the approved protocol.

### Reagents

CDDO was obtained from the National Cancer Institute, Bethesda, MD through the Rapid Access to Intervention Development Program

### Mice

TRAMP mice were bred by the Jackson Laboratories (Bar Harbor, ME, USA) through their Speed Expansion Service involving *in vitro *fertilization of C57BL/6 females with C57BL/6-Tg (TRAMP) 8247NG/J males. Mice were genotyped for the transgene (Tag) and delivered to us when 4 weeks old. Mice were maintained in temperature-controlled room (68-72°F) with a 12 h light/dark cycle and provided semi-purified AIN-76A mouse chow and water *ad libitum*. Mice were acclimated for one week before starting the experiment. All animal treatments were according to the protocol approved by the Institutional Animal Care and Use Committee [15].

### Treatment protocol

At the age of 5 weeks, 50 male TRAMP mice were weighed and randomized into two groups: Mice in vehicle control group (n = 25) were administered 0.1 ml of vehicle consisting of cremophor-EL:DMSO:PBS (1:1:8), 5 days a week for 20 weeks by p.o. gavage. In the treatment group (n = 25), mice were administered CDDO at a dose of 10 μmol/kg in 0.1 ml of vehicle, 5 days a week for 20 weeks. Body weight of control and CDDO-treated mice was recorded each week and mice were observed for treatment related stress, such as water and food withdrawal, unusual posture, ruffled fur or listlessness. Ten randomly selected animals from each group underwent *in vivo *MRI of prostate glands and seminal vesicles, just before (5 weeks of age), in the middle (at 12 weeks of age) and at the end of treatment. Same selected animals under went MRI at all time points, however, identity of individual animal was not tracked during imaging or at euthanasia due to loss of body markings or ear tags during the long treatment. At the age of 25 wk following MRI, mice from both groups were sacrificed 24 h after the last administration of vehicle or CDDO. After opening the abdomen, mice were visually observed for the presence of tumor mass, enlargement of seminal vesicles, prostate lobes, and pelvic lymph nodes. Urogenital tract and pelvic lymph nodes were removed and weighed. Tissue samples were processed for histological analyses.

### In Vivo MRI Studies by 3 Tesla

An appropriate state of anesthesia was obtained with isoflurane (3% for induction, 0.7% to 1.5% for maintenance mixed with O2). MRI was obtained with a 3 Tesla clinical MRI system (Signa Excite, GE health) using 50 mm diameter × 108 mm RF rung length small animal imaging coil (Litzcage small animal imaging system, Doty Scientific Inc, Columbia, SC). After positioning using a triplanar FLASH sequence, T1, and fast spin echo (FSE) fat suppression (FATSAT) T2-weighted images were obtained. T1- weighted images were obtained using following sequence; TR/TE = 300/10 ms, 128 × 128 matrix, 13-15 slices, 0.9 mm thick, 40 mm field of view (FOV), number of excitation (NEX) = 2-4. Fast spin echo T2-weighted images were obtained using standard two-dimensional Fourier transformation (2DFT) multislice (13-15) multiecho (2 echoes) MRI. A series of 2 sets of images (13-15 slices for each set) were obtained using TEs of 11 to 70 msec and a TR of 4000-5000 ms. The images were acquired using 40 mm FOV, 1 mm slice thickness, 128 × 128 matrix, and NEX = 2. We used clinical 3T MRI system due to availability of small animal coil synchronized with 3T MRI and optimized for small animal scanning. Moreover, all the available clinically approved MRI sequences can be used, especially fat suppression during T1 and T2-weighted images. Easy sequence manipulation and shorter acquisition time were also considered as advantages.

### Image analysis

**Figure 1:**
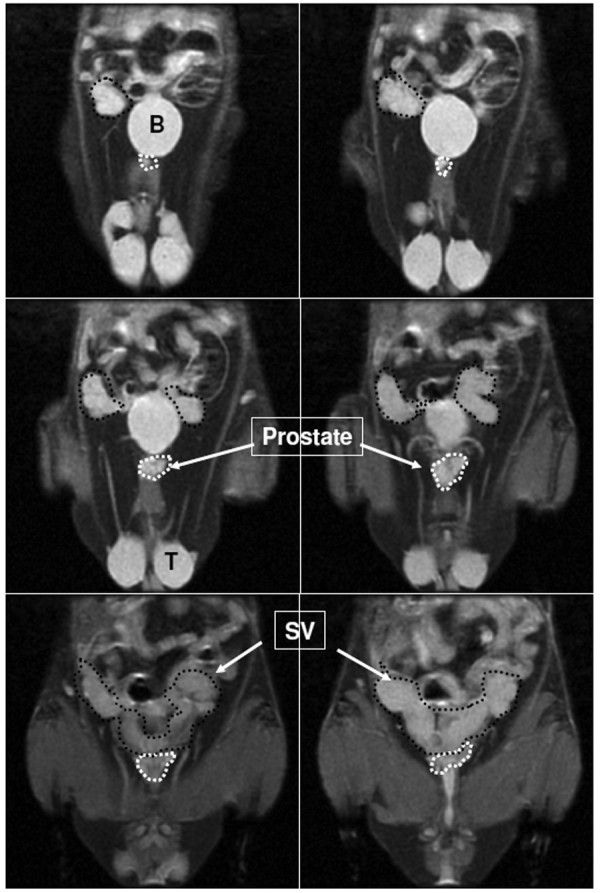
**Prostate gland and seminal vesicles on MR images**. Identification of prostate gland (areas inside white dots) and seminal vesicles (areas inside black dots) on fat saturation T2-weighted images. Volume of the prostate gland and seminal vesicles was determined by drawing irregular region of interest. B = urinary bladder, T = testes and SV = seminal vesicle.

All images were analyzed using our home made image analysis software "Eigentool" (http://www.radiologyresearch.org/eigentool.htm). Volume of the prostate glands and seminal vesicles were determined by an investigator blinded to the assignment of animals (treated vs control). The investigators were trained to indentify the prostate glands and the seminal vesicles by going through both axial and coronal images obtained with T1W and T2W sequences. Irregular region of interests (ROIs) were drawn over prostate and seminal vesicles separately and the areas were recorded (Figure 1). All the image sections that contained prostate and seminal vesicles were included in the analysis. Total calculated areas were multiplied by image thickness to determine the volume of prostate and seminal vesicles. The values were expressed in cm^3^. The volume of prostate gland calculated from MRI images was compared with the weight of the prostate gland (gm) determined at necropsy. MRI could detect dorsal and anterior/ventral lobes of the prostate gland clearly. However, due to partial volume effect the lateral lobes that continue with coagulating gland could not be separated from surrounding tissues and seminal vesicles. On the other hand, *ex vivo *extracted prostate glands comprised of all the lobes. It is expected that due to differential density between control (due to tumors) and treated animals the volume measured by MRI will not exactly match the weight of the extracted prostate glands and seminal vesicles.

### Analysis of signal intensity

According to Dr. Bradley (http://www.e-radiography.net/mrict/fund mr2/fundmri 2.htm) mathematically the intensity (I) of the spin echo signal can be approximated:

$$\text{I = N(H)f(v)(1 - }{\text{e}}^{\text{ - TR/T1}}\text{)}{\text{e}}^{\text{ - TE/T2}}$$

where N(H) is the NMR-visible, mobile proton density and f(v) is an unspecified function of flow. This equation indicates that the intensity of the MR signal increases as hydrogen density and T2 increase and as T1 decreases. It should also be noted that T1 and T2 influences are both subject to TR (repetition time) and TE (echo time). Thus, the effect of the T1 and T2 relaxation times of the substance on signal intensity is subject to the specific values of TR and TE selected before the image is acquired. When considered in the most simplistic terms, the spin echo is a two-step process. The first step (longitudinal recovery) determines the starting intensity for the second step (transverse decay). The starting intensity reflects the relationship between T1 and TR and is ultimately limited by the proton density. The subsequent decay from this starting intensity reflects the relationship between T2 and TE. As the TR is prolonged, all substances eventually recover full longitudinal magnetization between repetitions and the pixel intensity becomes dependent only upon proton density and is independent of T1. Substances with longer T2 times will generate stronger signals than substances with shorter T2 times, if both are acquired at the same TE and if proton density and T1 are comparable. Increasing the echo delay time (TE) increases the differences in the T2 decay curves between substances, increasing the T2-weighting. Images obtained with a sufficiently long TR and TE will cause fluid (such as CSF) or edema more intense than solid tissue (such as brain).

**Figure 2:**
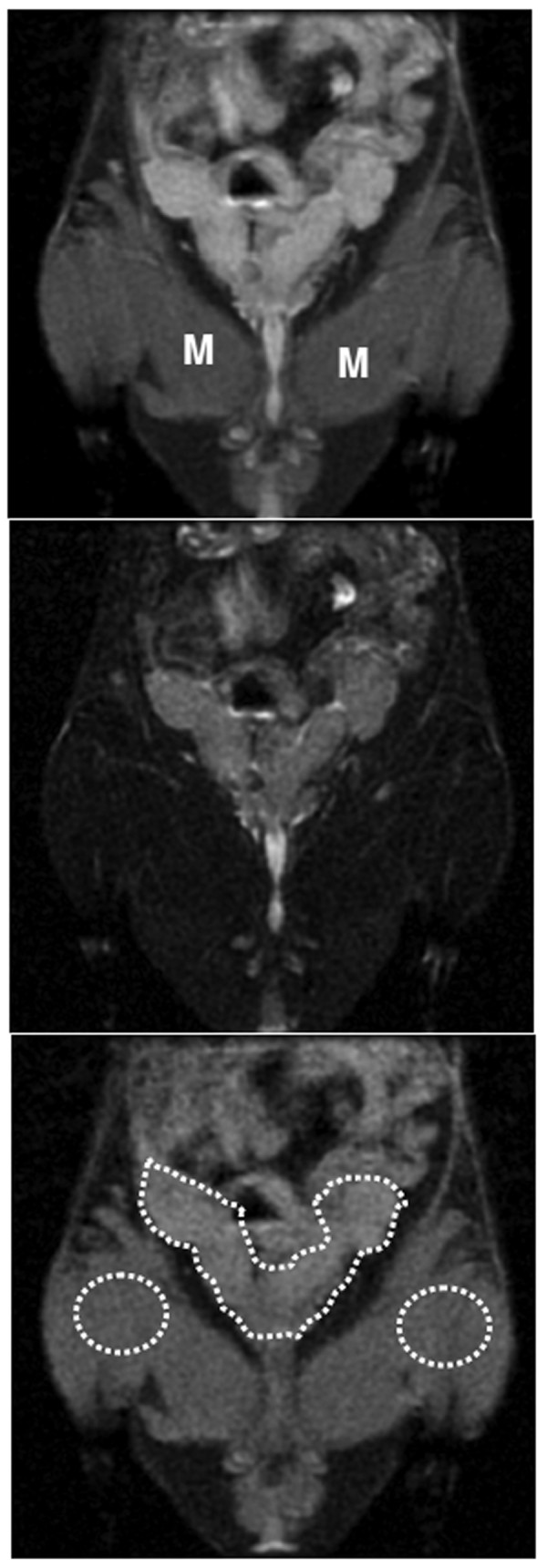
**Measurement of signal intensity changes**. Representative images of seminal vesicles from a mouse showing signal intensity on FATSAT T2-weighted image with a TE of 11 ms (upper), with a TE of 70 ms (middle) and subtracted image (lower). Note the differential signal intensity among different tissues on higher TE. ROIs on lower image indicate the representative areas from where signal intensity changes were determined. M = muscles.

It is expected that multiple small tumor foci and associated edema will cause lengthening of T2 values in seminal vesicles of control untreated animals compared to that of treated animals, therefore, signal intensity on T2-weighted images will vary based on the echo delay time (TE). The TR used in image acquisition is long enough to recover full longitudinal magnetization between repetitions and the signal intensity on MR images with different TE will be different between areas of normal seminal vesicle and in areas of tumor growths due to varying T2 values. We expect that tissues with higher T2 will show relatively high signal intensity on images obtained with higher TE and subtraction of the images of higher TE from lower TE will give differential image. To calculate the changes in signal intensities, images with higher TE (70 ms) were subtracted from corresponding images with lower TE (11 ms) and the resultant images were analyzed. The same investigator blinded to the assignment of animals drew ROIs on the left and right seminal vesicles (to include all areas of seminal vesicles in all sections) and on the skeletal muscles of the corresponding left and right thighs (Figure 2). Average signal intensities of seminal vesicles were normalized with corresponding average signal intensity of thigh muscles. It is to be cautioned that inflammation or abscess in seminal vesicles may give similar signal intensity changes, however, TRAMP model creates spontaneous small tumor foci in the seminal vesicles, and inflammation or abscess is not expected.

### Histological analysis

Tissue specimens of prostate gland and seminal vesicles for all animals that underwent MRI were fixed in 10% neutral buffered formalin for 48 h and then embedded in paraffin. Five to six micrometers thick sections were cut and stained with H&E for routine histology. Tumors were graded according to our published method in prostate glands in all control and treated animals [16] that underwent MRI by using photomicrographs from 5 randomly selected areas. Presence or absence of tumor foci in seminal vesicles was also checked and photomicrographed. For dorso-lateral prostate (DLP), histopathological classification was as follows; normal DLP (ducts lined with single layer of secretory epithelial cells surrounded by 2-3 cell layers of fibromuscular stroma); low-grade prostatic intraepithelial neoplasia (PIN) (epithelial cells with variably elongated nuclei with condensed chromatin); high-grade PIN (epithelial stratification and tufting, presence of micropapillary and cribiform structures); well differentiated (WD) carcinoma (epithelial cells invading fibromuscular stroma) and moderately (MD) to poorly differentiated (PD) adenocarcinoma of the prostate (sheets of neoplastic cells with little or no glandular structures).

### Data analysis

All data were expressed as mean ± one standard deviation. Either two way analysis of variance (ANOVA) or multivariate ANOVA followed by post-hoc test Fisher's PLSD was applied to determine the significant differences between treated and control or among different time points (5 weeks, 12 weeks and 25 weeks). A p-value of <0.05 was considered significant.

## Results

### Treatment with CDDO is well tolerated

Treatment with CDDO for 20 weeks was well tolerated without evidence of any noticeable toxicity with respect to animal appearance, behavior or change in the body weight. Body weight increased as a function of age at the same rate in both the vehicle control and treatment group from 20 ± 0.8 g (control group) and 20.5 ± 0.9 g (treatment group) at 5 weeks of age to 32.7 ± 1.5 g and 31.9 ± 1.9 g, respectively at 25 weeks of age.

### Prostate and seminal vesicles by in vivo imaging

**Figure 3:**
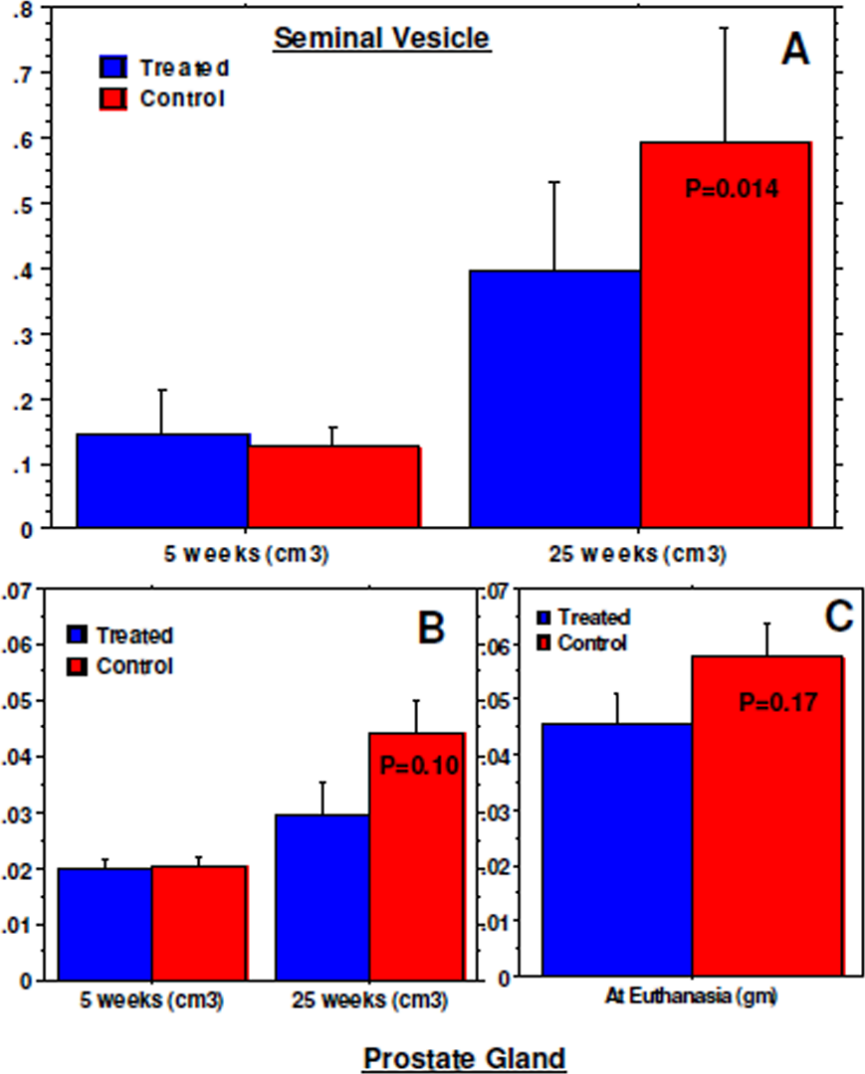
**Measurement of seminal vesicles and prostate gland volume by MRI**. (A) MR images show significantly increased volume of seminal vesicles by 25 weeks in control animals compared to that of treated animals. Note the similar volume at the beginning of the treatment (at 5 weeks). (B) Similar to seminal vesicles, prostate glands also showed higher volume in control animals on MRI which was also confirmed at necropsy (C, although significant differences were not achieved). Note the similar volume at the beginning of the treatment (at 5 weeks).

MR images were obtained before and after the start of the treatment. The analysis of images showed similar volume of seminal vesicles in control mouse (0.126 ± 0.03 cm^3 ^at 5 weeks) compared to that of treated (0.143 ± 0.07 cm^3 ^at 5 weeks) at 5 weeks. However the volume became significantly higher (p < 0.05) in control mice (0.596 ± 0.17 cm^3 ^at 25 weeks) compared to that of treated mice after 20 weeks following the start of the treatment (0.397 ± 0.13 cm^3 ^at 25 weeks) (Figure 3A). On the other hand, volumes of the prostate glands measured from 10 animals in each group were not different among the control (0.02 ± 0.006 cm^3^) and treated (0.02 ± 0.005 cm^3^) animals at 5 weeks. Increased volume of prostate gland was observed in control animals (0.044 ± 0.019 cm^3 ^at 25 weeks) compared to that of treated animals (0.03 ± 0.018 cm^3 ^at 25 weeks), although significant difference was not achieved (p = 0.102) (Figure 3B). Similarly, weights of the prostate glands collected from these same animals that underwent MRI also did not show significant difference (p = 0.17) between the control (0.058 ± 0.014 gm at 25 weeks) and treated groups (0.045 ± 0.019 gm at 25 weeks). The measured volume of prostate glands was in agreement with the measured weight determined during collection of tissues following euthanasia (Figure 3C) when compared between controls and treated animals (tendency to increase volume and weight in control group).

### Analysis of signal intensity

**Figure 4:**
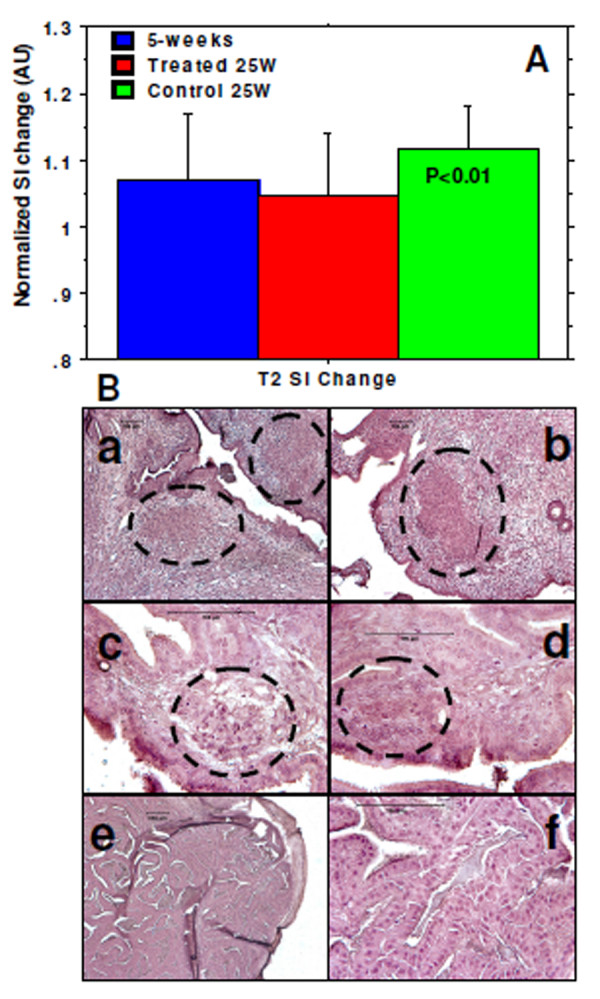
**Changes in signal intensity and micro-tumor foci**. (A) Changes in T2 signal intensity in seminal vesicles was significantly higher in control animals at 25 weeks, which indicate heterogeneous tissues (involvement of micro-tumor foci) compared to that of treated animals at 25 weeks or of animals at 5 weeks. (B) Multiple tumor foci were observed in seminal vesicles of control animals (a, b, c, d) compared to that of treated animals (e, f). a, b, e are with x10 and c, d, f are with x40 magnifications. Bars in all images represent 100µm.

Similar changes in signal intensity in seminal vesicle were observed in both groups of animals before starting the treatment at the age of week 5. However, when the signal intensity changes in seminal vesicles between the control and treated animals were compared at 25 weeks, significantly (p < 0.01) lower changes in signal intensity were observed in treated animals (Figure 4A), indicating less number of tumor foci to the seminal vesicles of treated animals. Control animals showed significantly higher changes in signal intensity in seminal vesicles at 25 weeks when compared with corresponding treated at 25 weeks (p < 0.001) and all animals at 5 weeks (p < 0.01). The MRI findings were also supported by histology analysis of seminal vesicles from two groups of animals (Figure 4B).

### CDDO slows down the progression of prostate carcinoma in TRAMP mice

**Figure 5:**
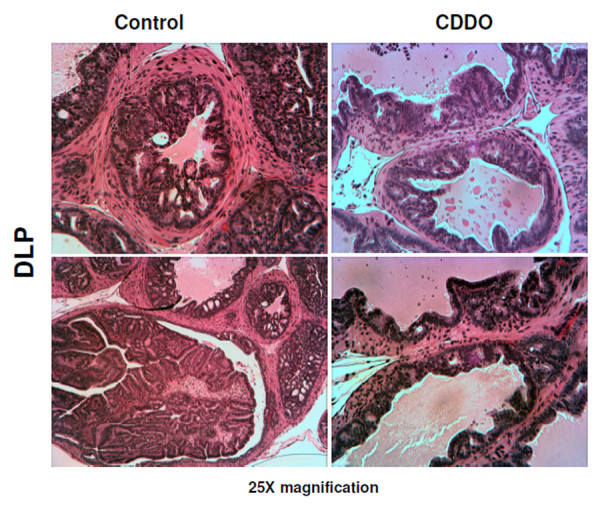
**HE staining of prostate gland**. H&E-stained sections of DLP from representative cases of 25 week old vehicle control or CDDO-treated TRAMP mouse. Control DLP showing well differentiated carcinoma and CDDO-treated DLP showing low-grade PIN. x25 magnifications.

In mice treated with CDDO for 20 weeks, two of the control mice (out of 25, did not undergo MRI) showed very large tumors originating from the prostate, whereas none of the CDDO treated mice showed these large tumors. In 7 out of 10 control mice (70%) tumor growth was evident in seminal vesicles as opposed to 3 out of 10 CDDO-treated mice (30%). There was no significant difference (p = 0.17) in the prostate gland weight when compared between the treated and non-treated animals that underwent MRI (n = 10 in each group). However, significant difference was noticed when all animals (n = 25 in each group) were combined (CDDO = 0.040.9 ± 0.013 gm; Control = 0.073.2 ± 0.014 gm, p < 0.01). Histological evaluation of the DLP of control mice (that underwent MRI) showed well-differentiated adenocarcinoma in 8 of the 10 mice (80%), 2 mice showed moderately differentiated CaP (20%), and none showed normal tissue, PIN or high PIN, indicating that 100% of the control mice had cancerous lesions in the DLP (Figure 5). In contrast, 1 CDDO treated mice (10%) had normal DLP, another showed well-differentiated CaP (10%), and 8 mice (80%) had PIN or high PIN lesions (that underwent MRI). These data demonstrated that majority of the treated animals (90%) had noncancerous lesions ranging from normal tissue to preneoplastic PIN and high PIN compared to 100% of the control mice that showed CaP in the DLP. CDDO also inhibited the progression of prostate cancer into the ventral prostate.

## Discussion

Although several murine prostate cancer models are available (e.g., LADY transgenic mine, PTEN null, MPAKT etc.), we used TRAMP mouse model in the present study because it is the most well-characterized and extensively used preclinical model of prostate tumorigenesis in chemoprevention studies. Probasin promoter directed expression of SV40 early genes in the prostate epithelium drives the development of prostate cancer in DLP and ventral prostate glands in an age-dependent manner beginning with the emergence of prostatic intraepithelial neoplasia (PIN), a preneoplastic lesion by 6-8 weeks of age followed by high grade PIN (HGPIN) by 10-12 weeks of age. Focal carcinoma develops between 12-18 weeks and poorly differentiated carcinoma is observed by the age of 18-24 weeks [17,18]. Tumor metastasizes to the liver, lungs, bone and pelvic lymph nodes by 24-30 weeks of age [19]. The slow progression of the multistep process of prostatic tumorigenesis over a period of 8-30 weeks renders TRAMP model highly suitable for exploiting the chemopreventive efficacy of novel agents. Indeed, TRAMP mice have been successfully used to evaluate the chemopreventive activity of several natural and synthetic compounds including sulforaphane, green tea polyphenols, garlic constituents, genistein etc. [13,20-23].

Most of the investigations that follow the progression of prostate cancer in chemopreventive treatment strategies use direct measurement of tumor weight/size as well as the weight of total genitourinary system as a measure of tumor progression following euthanasia. However, this method of measuring progression cannot follow the same animals over the span of the treatment duration. *In vivo *imaging modalities could be a great help to follow the same animals over the span of treatment. In our previous study, we have shown the importance of FATSAT T2-weighted MRI sequence in detecting prostate glands as well as orthotopic implanted prostate cancer [24]. One advantage of MRI is that the images of the prostate gland can be obtained in all planes to identify the glands correctly or MRI can be used to obtain 3D images to reformat later to see the gland in all planes (currently we are optimizing 3D isotropic image sequences). In the current study, we have also used FATSAT sequence to detect the prostate gland, although individual tumor was not detected clearly. Inability to detect the tumor in prostate gland could be due the model itself. TRAMP model produces micro tumors both in prostate and seminal vesicles unless it is in late stages of development. Histopathology also confirmed the size of the tumors in the gland and seminal vesicles. With the current hardware of 3T clinical MRI system, it may not be possible to detect these sub-millimeter size of prostate cancer by *in vivo *imaging. We need to have either high strength dedicated animal MRI system or imaging of animal in an *ex vivo *setting. Other technique would be to use contrast enhancement. We have not tried contrast enhancement in this study, although we think contrast enhancement may not give concrete results, knowing the size of the tumors. However, our *in vivo *imaging method has produced comparable results to that of measured prostate gland weight at necropsy. In that respect, our method of *in vivo *imaging can be used to follow the chemopreventive strategy in TRAMP model of mouse prostate cancer.

Designing an image analysis technique can indirectly show the presence or absence of tumors in any tissues. We have acquired the FATSAT T2-weighted images using two echoes (11 ms and 70 ms). Lower TE T2-weighted images usually indicate total density of the proton in a tissue, which could be almost identical throughout the tissue of interest, whereas high TE images usually produce differential signal intensity from a tissue/organ based on the differential relaxation or T2 parameters [25,26] (http://www.e-radiography.net/mrict/fund mr2/fundmri 2.htm). It is expected that multiple small tumor foci and associated edema will cause lengthening of T2 values in seminal vesicles of control untreated animals compared to that of treated animals, therefore, signal intensity on T2-weighted images will vary based on the echo delay time (TE). The TR used in image acquisition is long enough to recover full longitudinal magnetization between repetitions and the signal intensity on MR images with different TE will be different between areas of normal seminal vesicle and in areas of tumor growths due to varying T2 values. By subtracting high TE images from low TE images, we could have a differential signal intensity patterns which will be different between treated and control groups of animals. Our image analysis proved our hypothesis and there were significant differences in differential signal intensity between the treated and control animals at 25 weeks. Our image analysis findings were also supported by histopathology, where multiple microscopic tumor foci were observed in seminal vesicles of control animals.

## Conclusion

In conclusion, our study demonstrated the differences in the volumes of prostate gland and seminal vesicles in TRAMP mice following chemoprevention strategy and these changes can be detected by *in vivo *3T clinical MRI. *In vivo *MRI is helpful in assessing the chemoprevention of prostate cancer in TRAMP mice.

## Competing interests

The authors declare that they have no competing interests.

## Authors' contributions

ASA conceived the study and carried out MRI and involved writing the manuscript and trained AS to perform image analysis. AS carried out image analysis and data collection. NRSV helped MRI and histochemistry. DD and XG carried out treatment of the animals, maintenance and collection of tissue following euthanasia. AI maintained animals and prepare the histochemistry samples. BJ helped writing and discussion. MMA helped MRI and data analysis. SCG involved in over all management of the project and critical discussion regarding treatments. All authors read and approved the final manuscript.

## Pre-publication history

The pre-publication history for this paper can be accessed here:

http://www.biomedcentral.com/1471-2342/11/21/prepub
